# Analysis of Normalizers and Neurodevelopment-Related microRNAs in the Prefrontal Cortex and in the Sperm of SHR Rats

**DOI:** 10.3390/ijms262110589

**Published:** 2025-10-30

**Authors:** Isabelle Hernandez Cantão, Gabriella Mesas Campagnoli, Taiza Stumpp

**Affiliations:** Laboratory of Developmental Biology, Department of Morphology and Genetics, Federal University of Sao Paulo (UNIFESP), São Paulo 04023-900, SP, Brazil; isabellehcantao@gmail.com (I.H.C.); gabriella.campagnoli@unifesp.br (G.M.C.)

**Keywords:** schizophrenia, spontaneously hypertensive rat, miRNA, sperm, reference gene, prefrontal cortex, hippocampus

## Abstract

Schizophrenia (SCZ) is a psychiatric disorder that affects around 1% of the world’s population. Despite the large number of studies about SCZ etiology and heritability, a definitive and clear genetic basis for SCZ and its inheritance has not been established so far. Considering that SCZ is influenced by environmental aspects, the participation of epigenetic mechanisms in the development and manifestation of SCZ is considered. However, longitudinal clinical and molecular studies that follow SCZ development using brain tissue are unfeasible. Thus, animal models, such as the spontaneously hypertensive rats (SHR), have been used to explore some aspects of SCZ. In this study, we investigated the expression of miRNAs related to neurodevelopment and/or to SCZ in the brains and sperm of SHR rats via RT-qPCR. For this, a previous analysis of endogenous qPCR normalizers was performed. The results showed that miR-Let-7g seems to be a candidate endogenous normalizer for the brain and sperm. However, no alteration in the expression of SCZ-related miRNA was detected. These data indicate that further studies must be performed to address the applicability of the SHR model to study the miRNA related to SCZ and its paternal transmission.

## 1. Introduction

Schizophrenia (SCZ) is a psychiatric disorder that affects around 1% of the world’s population. It is characterized by emotional and consciousness issues that can lead to significant compromise of individual and familial welfare. Although SCZ shows 41% to 87% heritability, an established genetic diagnostic has not been confirmed, which explains the inclusion of SCZ among the complex and missing heritability disorders.

Studies about SCZ etiology have indicated that this disorder presents developmental aspects, such as an impairment of neurogenesis and of synapse establishment. To some extent, SCZ manifestation has been attributed to prenatal and childhood exposure to adverse conditions, such as maternal stress, malnutrition, trauma, and infections [1,2,3,4,5,6,7], which are conditions known to affect neurodevelopment. Alterations in the methylation [8] or insertions [9] of the retrotransposon LINE-1 have also been related to SCZ. This evidence indicates that the genetic aspects and environmental influences work together in SCZ manifestation and development. Environmental conditions influence the manifestation of phenotypic characteristics by acting through epigenetic mechanisms, including the action of short non-coding RNAs (sncRNA), leading to the modulation of gene expression. The sncRNAs are short (~22 to 29 nucleotides) regulatory RNA sequences that include microRNAs (miRNAs), PIWI-interacting RNAs (piRNAs), and short interfering RNAs (siRNAs), among others. Although most of these sncRNAs act to impede mRNA translation, some of them can also act at the pre-transcription level, promoting DNA methylation [10,11]. The importance of miRNA to brain development and physiology is clear, since around 70% of mammalian miRNAs have been identified in the brain [12]. Additionally, brain-specific miRNAs have been identified and associated with particular events of neurogenesis and brain development. The participation of sncRNAs, especially of miRNAs, in SCZ pathophysiology has been reported [13,14].

The study of psychiatric disorders in humans presents considerable challenges, especially when the aim is to obtain biological information from the brain. The study of conditions related to neurodevelopment is even more challenging, since a longitudinal epigenetic study of the individuals, from prenatal life until the manifestation of the symptoms, is not feasible so far. To try to circumvent some of these aspects, animal and in vitro models have been explored. The spontaneously hypertensive rat (SHR) has been emerging as a potential model for SCZ [15,16,17,18,19,20,21,22,23,24,25]. Previous studies showed that the SHR model shows psychotic-like symptoms, such as prepulse inhibition and hyperlocomotion, and negative symptoms, such as reduced social interaction, increased rearing behavior, and reduced social interaction [17,18]. Interestingly, these schizophrenia-like behaviors can be reduced by the administration of antipsychotic drugs [19].

As previously mentioned, the heritability of SCZ has been shown in 41% to 87% of the cases [26,27]. However, a genetic basis for this inheritance cannot be found in all cases, which indicates the participation of other mechanisms of inheritance. Studies have suggested and discussed that epigenetic inheritance may be responsible for the transmission of psychiatric disorders [28,29]. The transmission of stress-related behavior, caused by trauma experienced in different phases of life, to the next generations has been reported [30,31,32,33,34]. This suggests the existence of mechanisms of paternal epigenetic inheritance. Indeed, the transmission of epigenetic patterns via sperm has been reported (Rando, 2016 [35]; Stenz et al., 2018 [36]; Greeson et al., 2023 [37]), including miRNA alterations (Rodgers et al., 2015 [38]; Lee and Conine, 2022 [39]).

In this study, we investigated the expression of neurodevelopment-related miRNAs in the SHR brain and sperm. RT-qPCR is a standard technique to quantify miRNA expression. However, the analysis and validation of gene expression by RT-qPCR requires normalization by a reference gene, which is still a complicated issue when working with miRNAs, since an ideal reference endogenous miRNA has not been identified yet. Each type of tissue is expected to have its own specific reference genes, whose main characteristic is their stable expression level, even in different experimental conditions. So far, little information about adequate reference genes for the quantitative expression of miRNAs using PCR in rat sperm cells, the prefrontal cortex, and the hippocampus is available. Although the analysis of miRNA expression by RT-qPCR has been used, universal normalization for miRNA investigation using this technique has not been established so far, especially for small-scale analyses. Because of this, it is important to find adequate reference miRNA for each type of tissue/cell and condition. Thus, to investigate miRNA expression in the proposed SCZ model, we first searched for candidate reference miRNAs suitable for the samples used here. Here, we identified miRNA Let-7g as a good candidate reference miRNA that can be applied for the three types of samples analyzed, which allowed us to investigate the expression of miRNAs previously reported to be related to neurodevelopment and/or SCZ. Our findings suggest that, although SHR rats might indeed be an interesting model to study compartmental aspects of SCZ [24,25], further studies are necessary to understand the applicability of this model to investigate the miRNAs related to this disorder.

## 2. Results

### 2.1. Expression of the Candidate Reference Genes in the Brain and Sperm Cells

**Figure 1:**
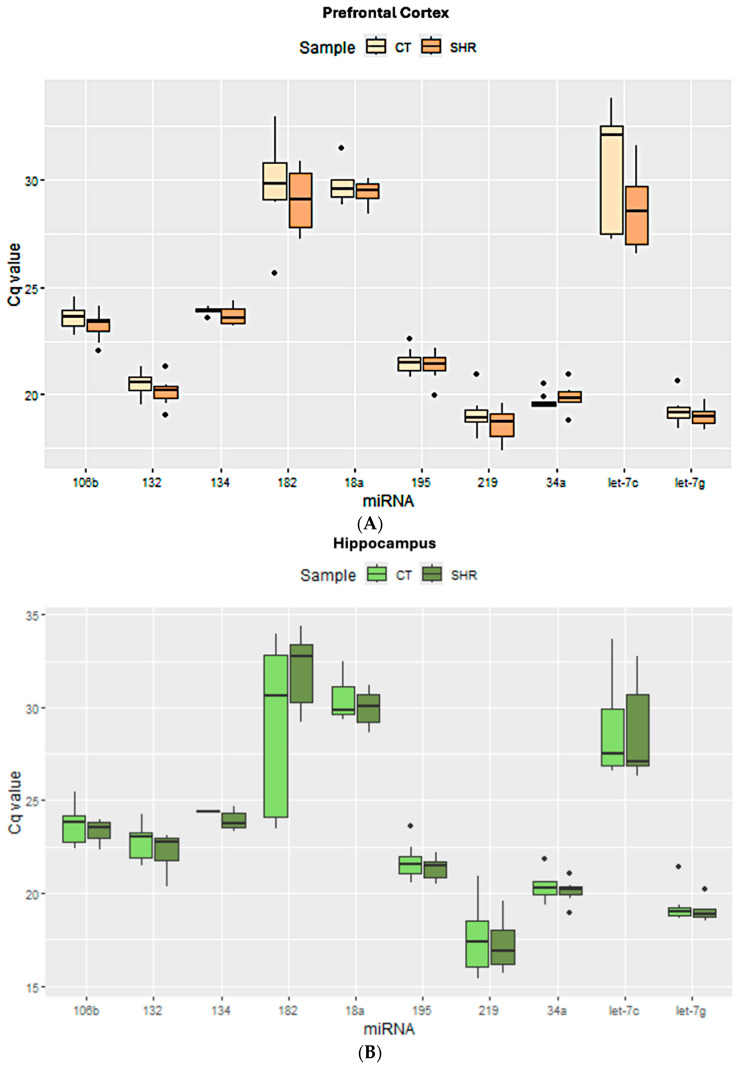

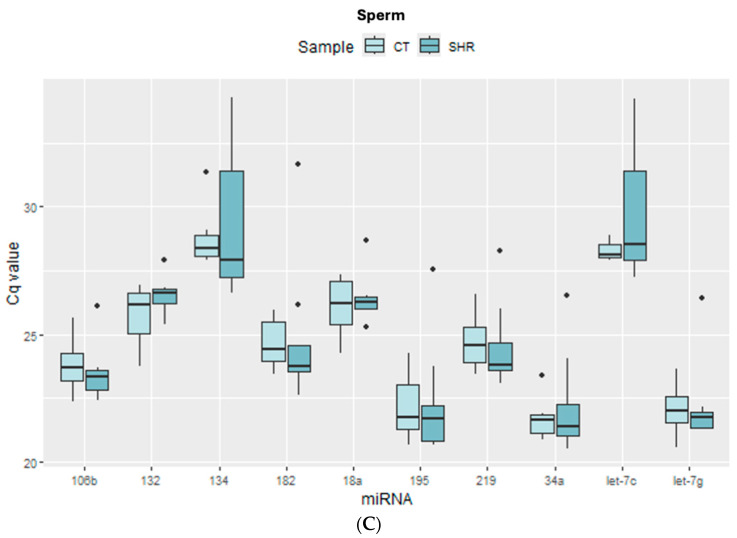
Cycle quantification (Cq) values of all target miRNAs obtained for prefrontal cortex (**A**), hippocampus (**B**), and sperm cells (**C**) from control and SHR groups.

Figure 1 shows the distribution of the cycle quantification (Cq) values for all miRNAs in the prefrontal cortex, hippocampus, and sperm. In the prefrontal cortex (Figure 1A) and in the hippocampus (Figure 1B), miR-219 and miR-Let-7g showed the lowest Cq mean, whereas in the sperm miR-34a, miR-195 and miR-Let-7g showed the lowest Cq mean (Figure 1C), both in the control and SHR groups, suggesting higher expression of these miRNAs in these samples. On the other hand, miR-134 showed the highest Cq mean in the sperm (Figure 1C), whereas miR-182 and miR-18a showed the highest Cq mean in the prefrontal cortex (Figure 1A) and hippocampus (Figure 1B), respectively, suggesting that the expression of these miRNAs is low in the respective cell or tissue.

The detection of miR-484 was very low in all tissues (Cq ≥ 35). Therefore, this miRNA was not included in the following analyses of candidate reference genes.

### 2.2. Reference Gene Analysis

To find the best candidate reference genes to study the expression of miRNAs in the sperm, prefrontal cortex, and hippocampus, two independent algorithms (Bestkeeper and NormFinder), as well as two algorithm-integrating programs (RefFinder and GenEx), were used, as previously mentioned. For each type of sample (prefrontal cortex, hippocampus, or sperm), the best candidate reference genes were indicated by each software, as shown in Table 1. When there were no common suggestions between the algorithms of the software, the two or three most pointed miRNAs were chosen for the analysis of miRNA expression (Section 4.3). A score has been established to show the candidate reference miRNAs that were most frequently indicated (Table 2). It is noteworthy that Let-7g appeared as a good candidate in all samples analyzed when samples and groups were analyzed altogether (control sperm + SHR sperm + control prefrontal cortex + SHR prefrontal cortex + control hippocampus + SHR hippocampus), as well as when the tissues were analyzed separately (Table 1 and Table 2). Indeed, from all miRNAs used in this study, Let-7g was the most stable miRNA among all samples and the one that showed less intragroup Cq variability (Figure 1). It suggests that, in the conditions of this study, this miRNA represents the best choice if one single reference gene is to be used for all samples and might be a potential wide-range reference gene for studies on miRNA expression in rats. Another important point to observe is that, in some cases, the software indicated different combinations of reference genes, depending on the integrating program (Table 1). This sheds light on the importance of using different independent and integrating algorithms to find more adequate reference genes.

### 2.3. miRNA Expression in Sperm, Prefrontal Cortex, and Hippocampus in Control and SHR Rats

**Figure 2:**
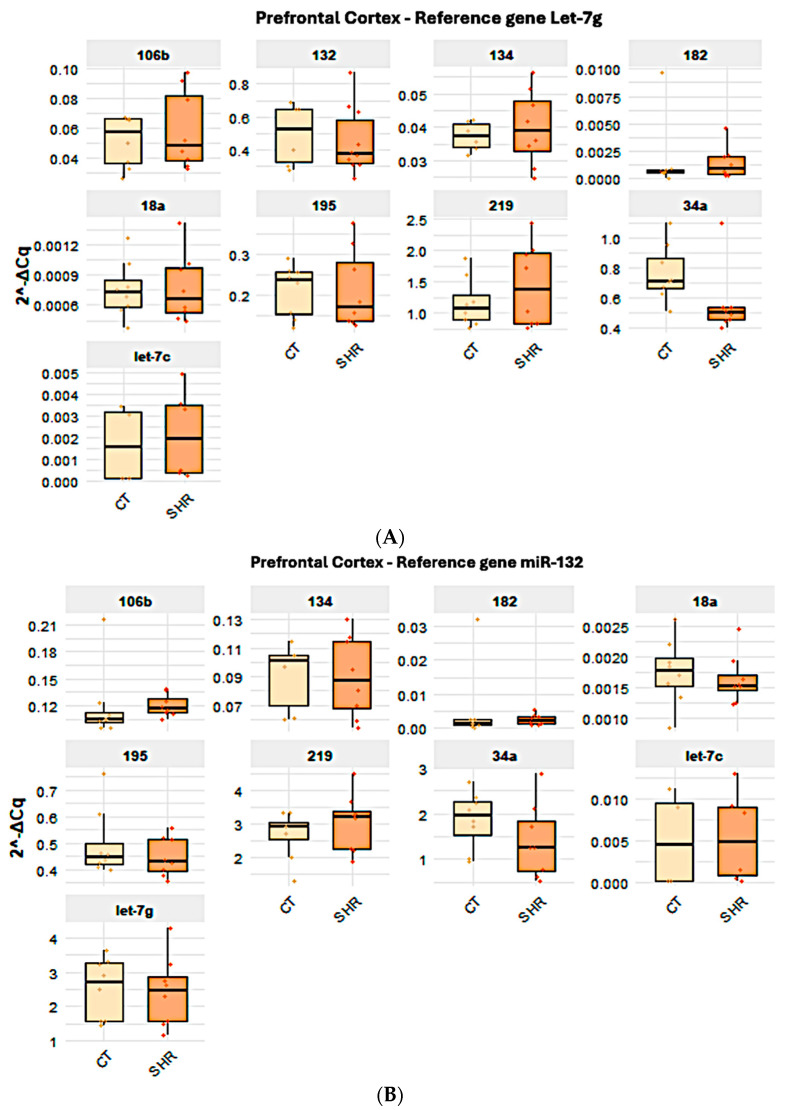

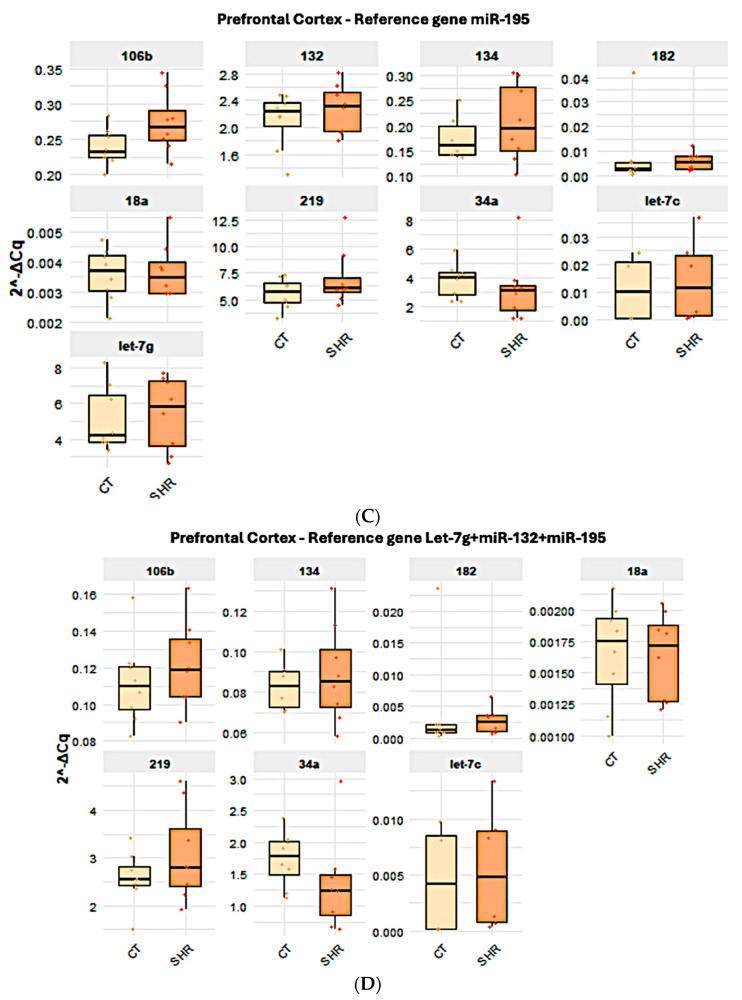
Expression of the target and reference miRNA in the prefrontal cortex of SHR and control rats using different candidate endogenous normalizers and their combinations. Regardless of the reference gene Let-7g (**A**), miR-132 (**B**), miR-195 (**C**), or Let7g + miR-132 + miR-195 (**D**), no difference in miRNA expression was observed.

**Figure 3:**
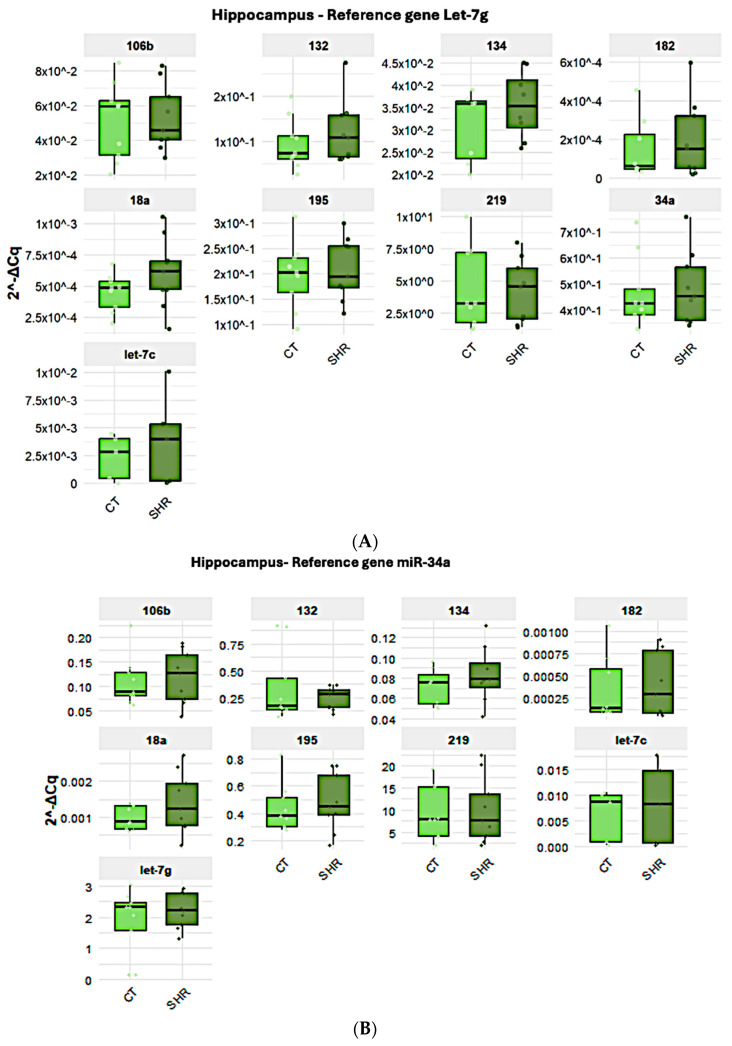

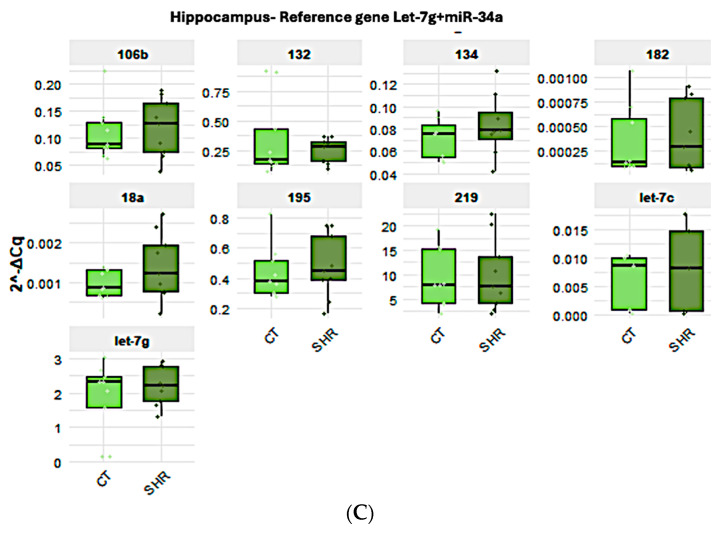
Expression of the target and reference miRNA in the hippocampus of SHR and control rats using different candidate endogenous normalizers and their combinations. Regardless of the reference gene Let-7g (**A**), miR-34a (**B**), or miR-34a + Let-7g (**C**), no difference in miRNA expression was observed.

**Figure 4:**
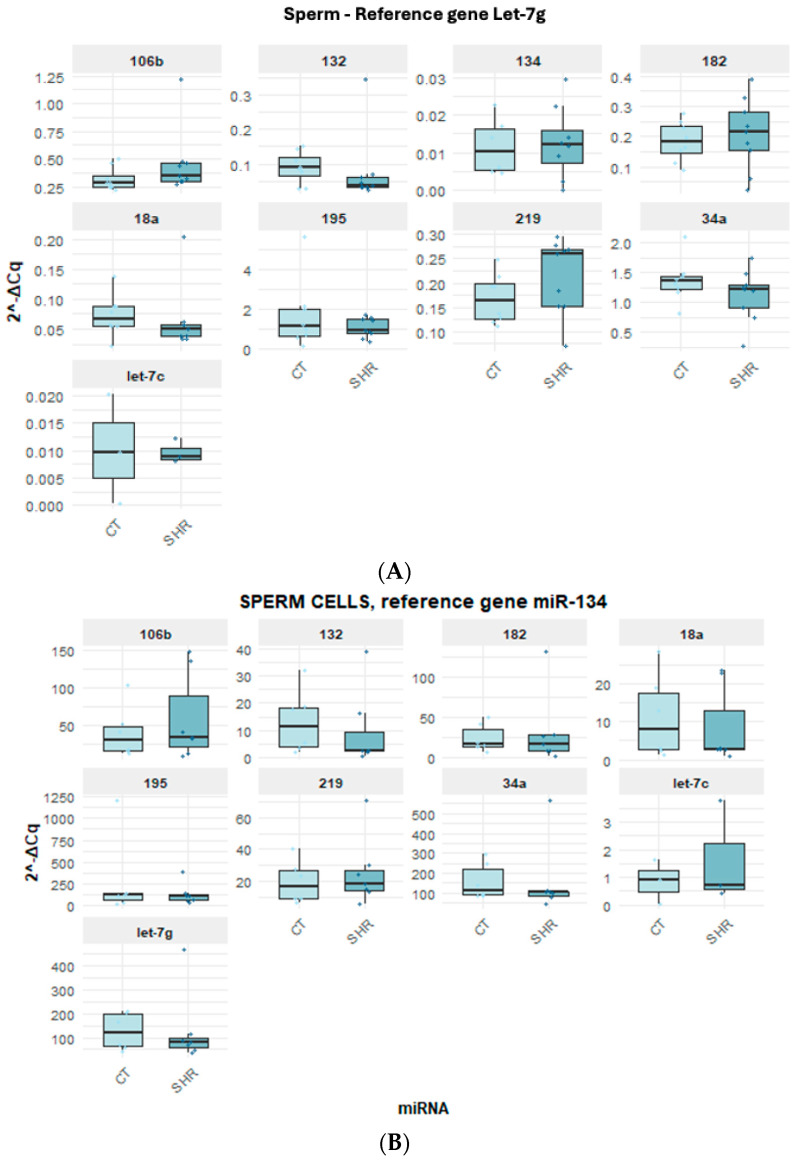

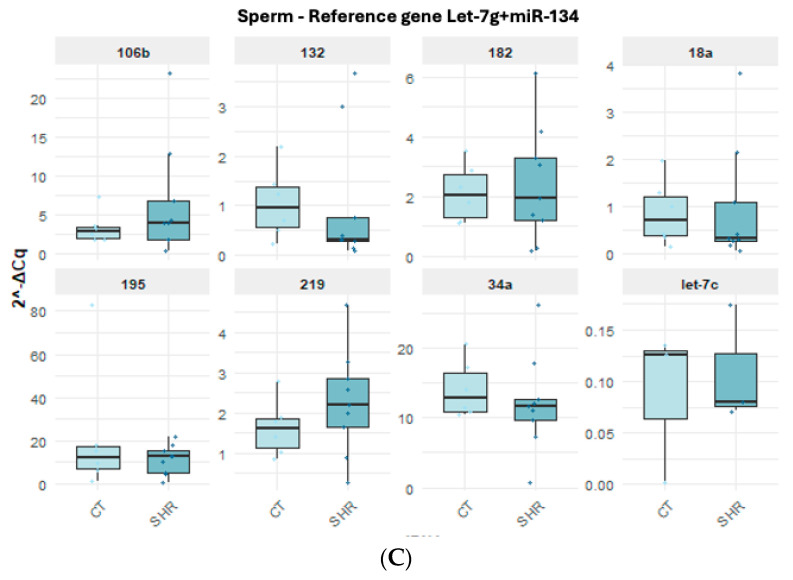
Expression of the target and reference miRNA in the sperm of SHR and control rats using different candidate endogenous normalizers and their combinations. Regardless of the reference gene Let-7g (**A**), miR-134a (**B**), or miR-134 + Let-7g (**C**), no difference in miRNA expression was observed.

All target miRNAs analyzed here were detected in the brain and in the sperm. However, the expressions of miR-18a and miR-182 were very low in the hippocampus and in the prefrontal cortex and are not represented here. When the expressions of the target miRNAs in the prefrontal cortex (Figure 2A–D), hippocampus (Figure 3A–C), and sperm (Figure 4A–C) were compared between the control and SHR rats, no statistically significant alteration in miRNA expression was observed in any of the samples (Figure 2, Figure 3 and Figure 4).

### 2.4. Effect of the Reference Gene Choice in the Analysis of the Expression of Target miRNA in Control and SHR Rats

As previously mentioned, different candidate reference genes, as well as different gene groups for each tissue/cell, were identified by the software. Also, most data indicated that Let-7g is the first choice when considering all three tissues/cells studied here. Indeed, the analysis of the expression levels of the target miRNAs in the control and SHR rats varied according to the reference miRNA chosen, but no differential expression was observed between the control and SHR groups, regardless of the reference miRNA chosen. The normalization of Let-7g expression by the other candidate reference genes in the sperm, prefrontal cortex and hippocampus revealed that Let-7g expression is higher in these samples (Figure 2, Figure 3 and Figure 4) than the other genes analyzed, as indicated by the 2^−ΔCq^ values, what agrees with the Cq mean values (Figure 1), except for miR-219 in the hippocampus (Figure 3A) and miR-34a in the sperm (Figure 4A). In general, Let-7g showed the lower intragroup variability of Cq in the sperm (Figure 1C) and hippocampus (Figure 1B), for both control and SHR groups. In the prefrontal cortex, however, Let-7g showed a slightly higher intragroup Cq variability in the control group than miR-34a and miR-195 (Figure 1), which might have led the software to identify these miRNAs over Let-7g in this tissue.

## 3. Discussion

The participation of miRNAs in the modulation of biological phenomena and disease has been the subject of a considerable diversity of studies. The importance of miRNA for nervous system development, physiology, and pathology, for example, has been clearly demonstrated. Elegant studies have shown that miRNAs are important for neurogenesis and synaptogenesis, as well as for synapsis plasticity and morphology. Thus, it is not surprising that miRNA dysregulation is involved in neurological conditions, such as psychiatric disorders.

One approach to explore miRNA roles in the nervous system and its related conditions is real-time PCR. However, one key challenge of using this technique to study miRNA expression is to find adequate endogenous controls for expression normalization. Thus, although studies about miRNA functions in the prefrontal cortex, hippocampus, and sperm are available, well-established endogenous reference miRNAs for RT-qPCR studies have not been found so far. The search for a good endogenous normalizer for the tissues/cells from both control and SHR rats used in this study proved to be mandatory. So, before analyzing possible differences in the expressions of neurodevelopment-related miRNAs between SHR and control rats, we searched for endogenous normalizers that could be suitable for both brain tissues and sperm cells.

The bioinformatic analysis using different software for reference gene definition showed that Let-7g and miR-34a were the best reference miRNAs when the three samples were combined. This result was surprising at first, since the brain and the sperm are very different from each other. On the other hand, it is possible that this similarity reflects the embryonic origin of the brain and the sperm, i.e., both differentiate directly from the epiblast. There are indications that the embryological origin can be traced by common miRNA expression. In line with this, miRNAs related to ectoderm-biased cellular states have been proposed [40]. More importantly, it has been shown that neurons and sperm share common biological characteristics, such as similar receptors and proteins, as well as signaling pathways [41]. Thus, it is reasonable to infer that there are miRNAs that are common to these two cell types. Indeed, a relationship between brain and sperm miRNA expression has been indicated in rats [42]. When the three types of samples (PFC, hippocampus, and sperm) were analyzed separately, these miRNAs still appeared amongst those with a high score. But differences were observed, although Let-7g showed a slightly lower variability in the sperm and in the prefrontal cortex than miR-34a.

The role of miR-34a has been shown in neurogenesis and neural differentiation [43,44,45,46]. In rats, the overexpression of this miRNA improves learning and memory consolidation and reduces emotionality [46]. Interestingly, the dysregulation of miR-34a has been reported in peripheral blood mononuclear cells [47,48] and in the pre-frontal cortexes [45] of individuals diagnosed with SCZ and has been suggested as a potential biomarker for SCZ diagnosis. Let-7g, in its turn, is a widely expressed miRNA that has been related to cancer and to schizophrenia as well [49,50]. The wide variety of processes in which this miRNA is involved counts positively towards its usefulness as a reference miRNA. On the other hand, this characteristic makes it important to confirm its suitability for each type of sample and specific condition. For example, in a previous study, we showed that supplementation with exogenous melatonin can lead to pronounced alteration of Let-7g expression in the brains and sperm of Wistar rats [42].

It is important to consider that there are commercially available exogenous miRNAs, obtained from plants or worms, that are recommended for differential expression analysis. However, these molecules must be introduced into the experiment at the moment of sample lysis. Depending on the type of experiment and sample availability, it is not possible to include the exogenous normalizers. Thus, the search for endogenous normalizers can be of use in specific conditions.

Despite the suggestions about the relationship between miRNA dysregulation and psychiatric disorders, studies about the molecular aspects of the brain from patients diagnosed with these disorders present obvious difficulties. Thus, the use of animal models has been presented as an important way of contributing to the knowledge in the field. Here, we investigated, in the SHR model, the expression of miRNA previously described as related to neurological processes or to SCZ. The SHR rat strain has been indicated as a valuable model to study the behavioral aspects of SCZ [15,16,17,18,19,20]. Despite this, our results showed that none of the eight target miRNAs analyzed here was differentially expressed in the brains or sperm of SHR rats. It was surprising, considering the neurological-related functions of these miRNAs and the SCZ-like behaviors reported in the SHR strain. However, it is important to consider that only two regions of the brain were analyzed. Thus, it is important to study other regions of the brain, such as the temporal lobe and the amygdala, to check whether these miRNAs are dysregulated in other areas of the brain that have also been implicated in SCZ. Also, it is valuable to consider that, although we did not find any statistically significant altered miRNA, it is important to consider that biological effects might be observed, even if the alteration is mild. miR-106b, for example, exhibited a *p*-value of 0.07 when prefrontal cortex and sperm samples of the SHR group were compared to CT samples. Thus, the possibility that the alterations of these miRNAs have consequences for brain function or the embryo derived from these sperm cells must be considered. Future studies are necessary to investigate this hypothesis.

Although no alteration in the expression of miR-34a has been detected in the sperm in this study, it is enriched in these cells when compared with the pre-frontal cortex and hippocampus in control Wistar rats, suggesting its relevance in sperm physiology and/or in early embryonic development. Indeed, previous studies showed that miR-34a regulates sperm motility in zebrafish [51] and is altered in the semen of infertile men [52]. In addition, the understanding that SCZ manifestation and development has a considerable environmental component has pointed to the participation of epigenetic mechanisms in SCZ etiology [53,54,55]. Interestingly, studies have shown that environmental aggression can cause epigenetic alterations in the brain and in the male gamete and that these alterations can be transmitted to the next generation and lead to psychiatric disorders, including SCZ [29,56]. A study showed that alteration of synaptic plasticity caused by stress in male rats can be transmitted to the descendants via sperm, through miRNA dysregulation [3].

It is interesting to mention that, although we did not find differential miRNA expression by RT-qPCR, a previous study of our group using next-generation sequencing (NGS) showed dysregulation of miR-106b in the sperm and prefrontal cortex of SHR rats [57]. This calls attention to the importance of using different strategies for biomarker investigation. Finally, the results obtained here suggest that it is important to extend the studies about endogenous normalization of miRNA expression in the brain and sperm, as well as about the miRNA profile in SHR models, since the literature points to this strain as a valuable model to explore behavioral characteristics of SCZ.

## 4. Materials and Methods

### 4.1. Animals and Sample Collection

Wistar rats (*Rattus norvegicus albinus*) and SHR rats (spontaneously hypertensive rats) obtained from the Centre for Development of Animal Models for Medicine and Biology (CEDEME) were used for this study. The adult animals were kept in plastic cages under a 12–12 h light/dark cycle at 23–25 °C. Food and water were allowed ad libitum. The animals were divided into two groups: control (CT), composed of Wistar rats (n = 9), and SHR, composed of SHR rats (n = 9). Euthanasia was performed by anesthesia/analgesia (xylazine/ketamine, 10 mg/kg and 100 mg/kg, respectively) followed by cardiac incision. During anaesthesia/analgesia, the sperm, the prefrontal cortex, and the hippocampus were collected and submitted to microRNA isolation, as described later.

### 4.2. MicroRNA Extraction, cDNA Synthesis, and qPCR

MiRNAs were isolated from the sperm, prefrontal cortex, and hippocampus samples using the mirVana^®^ miRNA Isolation Kit (AM1560—Thermo Fisher Scientific, Waltham, MA, USA). MiRNA quantification was performed by the Bioanalyzer (Agilent, Palo Alto, MA, USA) using the Small RNA Kit (Cat. 5067-1548, Agilent). cDNA was synthesized using the TaqMan Advanced miRNA cDNA Synthesis kit (Thermo Fisher Scientific—A28007). For all kits, the protocols indicated by the manufacturer were followed. Quantitative PCR was performed using custom TaqMan miRNA Fast Advanced assays (Thermo Fisher Scientific—4444556). Ten miRNAs were chosen based on the literature and on the Thermo Fisher Scientific website (Table 3). For the sperm, the SpermBase was also consulted (http://spermbase.org/ (accessed on 1 February 2023)). Since we intended to investigate how suitable the SHR strain could be for studies about schizophrenia, we chose miRNAs related to neural development and function, and that have been suggested to be involved in SCZ: miR-18a [58], miR-34a [13,45,47,59,60,61,62,63], miR-106b [64,65,66], miR-132 [67,68], miR-134 [68], miR-182 [69,70], miR-195 [49,71], miR-219 [49,72,73], and miR-484 [74,75]. In addition, because we intended to find candidate reference miRNAs for this study, members of one of the largest and most conserved families of miRNAs across different species were also included (Let-7g and Let7c) [76]. The assays used are described in Table 3. qPCR reactions were performed using the Roche LightCycler 96 (Roche, Basel, Switzerland) platform.

### 4.3. Reference miRNA Analysis for qPCR Normalization

Currently, there is no well-established endogenous control for the normalization of miRNA analysis via RT-qPCR. For this reason, this analysis was performed to support the analysis of miRNA expression in this study. The samples were separated into two groups: SHR and control (CT), according to each cell/tissue (sperm, prefrontal cortex, or hippocampus). The cycle quantification (Cq) values were input into the following software to obtain candidate reference miRNA: NormFinder (version 0.953; https://www.moma.dk/software/normfinder (accessed on 1 February 2023)) [55], RefFinder (https://www.ciidirsinaloa.com.mx/RefFinder-master/ (accessed on 1 February 2023)) [74], and GenEx (http://genex.gene-quantification.info/ (accessed on 1 February 2023)).

NormFinder is an algorithm that provides candidate reference genes based on the expression stability of the inputted genes and considering the intra- and intergroup variations, i.e., the best candidate reference gene is the one that shows a lower variation in the expression levels among the groups and the individuals [75,76].

GenEx incorporates the geNorm and NormFinder algorithms. As referred to in NormFinder, GenEx also indicates the best candidate reference gene based on the stability of the expression of the provided genes. Finally, RefFinder integrates geNorm and NormFinder, as does GenEx, but also includes BestKeeper and the comparative ΔCq method. RefFinder considers the geometric means of the weights attributed to each individual gene from the pool of genes provided. [75,76]. A score was established to represent the most frequently indicated candidate reference for each tissue/cell, as well as for all tissues/cells together. Since nine independent or integrated algorithms were used, miRNAs that appeared 8 to 9 times were scored with five stars, those that appeared 6 to 7 times received four stars, the ones with 4 to 6 appearances received three stars, and those that appeared 3 times received two stars.

### 4.4. RT-qPCR

For the analysis of the expression of the target psychiatric disorder-related miRNAs, the ∆Cq was calculated for each gene and each animal individually. The ΔCq values were obtained by the difference between the Cq of the target genes and the selected reference genes for each tissue. The values were log-transformed to obtain the relative expression of the target miRNA in the SHR and CT groups. The same miRNA input was used for all individuals to perform cDNA synthesis. Likewise, the same cDNA input from all of them was used to perform the qPCR assays.

### 4.5. Analysis of Predicted Target mRNAs

The Targetscan and miRDB miRNA databases were used as tools to investigate the predicted or experimentally confirmed target mRNA for miR-18a, miR-34a, miR-106b, and miR-134. For each of the two databases, hundreds of targets were obtained. We focused on those that appeared in both databases and species (rat and human) and have already been described as related to schizophrenia [77,78,79,80] or to any aspect of the central nervous system development or physiology.

### 4.6. Statistical Analysis

The data distribution was previously analyzed by the Shapiro–Wilk test. The Wilcoxon non-parametric test was applied when the data did not show normal distribution, and the *t* test was used when normal distribution occurred. The differences were considered statistically significant when *p* ≤ 0.05.

## 5. Conclusions

The data obtained in the present study shows that miRNA Let-7g might be an interesting endogenous normalizer for RT-qPCR studies on miRNA expression in brain and sperm samples. We also suggest that the neurodevelopment-related miRNAs explored here do not reflect the SCZ-like behavioral characteristics observed by others in the SHR strain, indicating that further studies must be carried out to investigate the suitability of the SHR model for epigenetic studies about SCZ.

## Figures and Tables

**Table 1:** Best reference genes for each tissue or cell according to each software.

	GenEx (V. 7)		RefFinder (V. 1)					Bestkeeper (V. 1)	NormFinder (V. 0.953)
	NormFinder	Genorm	RefFinder	ΔCt Method	Bestkeeper	NormFinder	Genorm		
All tissues	Let7g	miR-34a	miR-Let7g	miR-Let7g	miR-195	miR-Let7g	miR-34a	miR-34a	miR-Let7g
	miR-34a	miR-Let7g	miR-34a	miR-34a	miR-34a	miR-34a	miR-Let7g	miR-Let7g	miR-34a
Sperm	Let7g	miR-34a	miR-Let7g	miR-Let7g	miR-219	miR-Let7g	miR-34a	miR-219	miR-Let7g
	miR-219	miR-132	miR-34a	miR-34a	miR-Let7g	miR-219	miR-132	miR-132	miR-219
PFC	miR-34a	miR-132	miR-195	miR-219	miR-132	miR-34a	miR-195	miR-195	miR-34a
	Let7g	miR-195	miR-219	miR-Let7g	miR-195	miR-Let7g	miR-219	miR-132	miR-132
HIPPO	miR-34a	miR-34a	miR-195	miR-Let7g	miR-195	miR-34a	miR-195	miR-34a	miR-34a
	miR-132	miR-Let7g	miR-Let7g	miR-195	miR-34a	miR-132	miR-Let7g	miR-Let7g	miR-132

**Table 2:** Reference gene score.

	miRNA	Score
All samples combined	Let7g	★★★★★ *
	miR-34a	★★★★★
Sperm	Let7g	★★★★
	miR-219	★★★
	miR-34a	★★
	miR-132	★★
PFC	miR-195	★★★
	Let7g	★★
	miR-34a	★★
	miR-219	★★
	miR-132	★★
HIPPO	miR-34a	★★★★
	Let-7g	★★★
	miR-132	★★
	miR-195	★★

* The number of stars indicates how suitable each miRNA is as a reference for each sample type, where five stars indicates greater suitability.

**Table 3:** Selected miRNAs and their respective assays.

Gene	Assay Name	Assay ID	miRNA Sequence
miR-132-5p	rno-miR-132-5p	rno481320_mir	ACCGUGGCUUUCGAUUGUUACU
miR-195-5p	rno-miR-195-5p	rno480882_mir	UAGCAGCACAGAAAUAUUGGC
miR-34a-5p	rno-miR-34a-5p	rno481304_mir	UGGCAGUGUCUUAGCUGGUUGU
miR-219a-5p	rno-miR-219a-5p	rno481348_mir	UGAUUGUCCAAACGCAAUUCU
miR-134-5p	rno-miR-134-5p	rno480922_mir	UGUGACUGGUUGACCAGAGGGG
miR-18a-5p	rno-miR-18a-5p	rno480968_mir	UAAGGUGCAUCUAGUGCAGAUAG
miR-106b-5p	rno-miR-106b-5p	rno478412_mir	UAAAGUGCUGACAGUGCAGAU
miR-182	rno-miR-182	rno480960_mir	UUUGGCAAUGGUAGAACUCACACCG
miR-let-7g-5p	hsa-let-7g-5p	478580_mir	UGAGGUAGUAGUUUGUACAGUU
miR-484	hsa-miR-484	478308_mir	UCAGGCUCAGUCCCCUCCCGAU

## Data Availability

All protocols are fully described in the manuscript. No restrictions to raw qPCR data (Cqs) and bioinformatics results apply.

## Abbreviations

The following abbreviations are used in this manuscript:

SCZ	Schizophrenia
SHR	Spontaneously hypertensive rats
miRNA	microRNA
RT-qPCR	Reverse transcription—quantitative polymerase chain reaction
LINE-1	Long Interspersed Nuclear Element-1
sncRNA	Short non-coding RNA
piRNA	PIWI-interacting RNA
siRNA	Small interfering RNA
Cq	Cycle quantification
